# Time Space Translation: A Hox Mechanism for Vertebrate A-P Patterning

**DOI:** 10.2174/138920212800793375

**Published:** 2012-06

**Authors:** AJ Durston, S Wacker, N Bardine, HJ Jansen

**Affiliations:** 1 Institute of Biology, University of Leiden, Sylvius Laboratory, Wassenaarseweg 72, 2333 BE, Leiden, Netherlands; 2 Institute of Biochemistry, Albert- Einstein-Allee 11, University of Ulm, 89081, Ulm, Germany

**Keywords:** Colinearity, Gastrulation, Hox, Metagene, Time Space Translation, Xenopus.

## Abstract

The vertebrate A-P axis is a time axis. The head is made first and more and more posterior levels are made at later and later stages. This is different to the situation in most other animals, for example, in Drosophila. Central to this timing is Hox temporal collinearity (see below). This occurs rarely in the animal kingdom but is characteristic of vertebrates and is used to generate the primary axial Hox pattern using time space translation and to integrate successive derived patterns (see below). This is thus a different situation than in Drosophila, where the primary pattern guiding Hox spatial collinearity is generated externally, by the gap and segmentation genes.

## INTRODUCTION: THE HOX GENES

The Hox genes are major regulators of the animal bodyplan. This family of genes for transcription factors is involved in determining the identities of sequential zones along the main body axis. The Hox genes are contained in genomic complexes, containing up to 14 genes and these complexes show collinearity – spatial and sometimes temporal ordering of the expression of the Hox genes, corresponding to their 3’ to 5’ genomic order in a Hox complex. This is a spectacular phenomenon that has excited life scientists since it was discovered. See Figs. (**1** and **2**). We concentrate here on a Hox dependent mechanism that is involved in generating the main A-P axial pattern in vertebrates.

## HOX GENES AND THE VERTEBRATE A-P AXIAL PATTERN

How is the main vertebrate A-P hox pattern generated? This is clearly a complex matter. One copy of the embryonic pattern is in the axial mesoderm, which generates the axial skeleton and in the paraxial mesoderm, and is generated in association with somitogenesis [4]. There is also a pattern that runs in parallel, in the developing central nervous system (CNS) and its derivatives, arising from the neural crest [5,6,7]. These patterns are clearly coordinated, though there is a phase shift. The CNS pattern is slightly anterior relative to the mesodermal pattern [8]. This phase shift between the patterns of the two germ layers presumably arises by differential growth or morphogenesis, after gastrulation, when these patterns are initially generated together. There are clearly many regulators, acting at different stages, to coordinate, maintain and modify these two patterns but there is an important common element that integrates the whole pattern. This is that the vertebrate A-P axis is a time axis [44]. The head is made first and more and more posterior levels are made at later and later stages. So posterior Hox genes start expression later and will need dominance to exert their functions. Central to this timing is Hox complex temporal collinearity, that is used to generate the primary spatially collinear Hox pattern and integrate all of the successive derived patterns. This is thus a different situation than Drosophila, where the primary pattern guiding Hox spatial collinearity is generated externally, by the gap and segmentation genes.

## ORIGIN OF THE VERTEBRATE A-P PATTERN DURING GASTRULATION

### The General Features

The primary vertebrate A-P axial pattern begins to be generated initially early in development, during gastrulation (see below). No specific A-P regulators are known that operate before this stage [9]. See Fig. (**3**). We will describe the situation in the Amphibian (Xenopus) embryo, where most is known. Other vertebrates seem to be similar.

The gastrula mesoderm acquires patterning information first and involutes into the embryo The movements involved appear to be regulated by chemotaxis (Chuai *et al*., this volume [59]). This mesoderm then copies its information to an adjacent tissue layer: the overlying neurectoderm, which develops most of the A-P pattern by the end of gastrulation. This copying process is called vertical signalling [10, 11]. The mesoderm copies A-P information onto the neurectoderm. Initial vertebrate A-P patterning correlates with the initial gastrula stage expression of the Hox genes. The timed patterning process clearly continues after gastrulation, perhaps because the gastrulation process continues in the chordaneural hinge in the vertebrate tailbud [12].

### Hox Genes,Temporal Collinearity

**Fig. (4). Temporal Collinearity And Time space translation.**
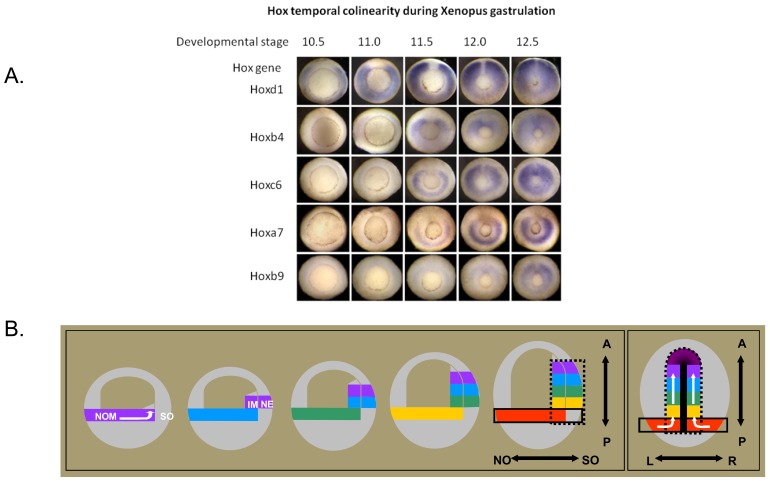
**a**. Temporal Collinearity In the Xenopus Gastrula. The figure [from 11] shows Hox expression patterns at sequential stages during gastrulation in Xenopus. The embryos are seen from underneath, where a ring (the blastopore) shows the position where mesoderm tissue invaginates during gastrulation. This ring gets smaller as gastrulation proceeds and the upper tissues in the embryo spread out and cover the lower part of the embryo (epiboly). The expression of several different Hox genes, seen as blue colour by in situ hybridisation, is in each case initially in the gastrula mesoderm in the zone above (outside) the ring. Hox expression is thus seen as a blue ring, and since it is initially only in part of the mesoderm (non organiser nesoderm), the ring is initially broken. This ring of Hox expression gets smaller as the blastopore ring gets smaller and mesoderm involutes into the embryo. The figure shows expression of a sequence of Hox genes with different paralogue numbers, between 1 and 9. It will be seen that the Hox gene with the lowest paralogue number starts expression first and later numbers start sequentially later. It will also be seen that the Hox genes in this time sequence include members of all of the 4 primary vertebrate paralogue groups (a,b,c,d). **b**. Time-space translation. Timed interactions between the Hox expressing non-organiser mesoderm and the Spemann organiser generate positional information during Xenopus gastrulation. The drawings [from 11] show simplified 2-dimensional representations of Xenopus gastrulae. The first 5 drawings show parasagittal (ventral to dorsal) two dimensional representations of gastrula profiles, starting at the beginning of gastrulation and then at sequential stages till the end. The last (6th.) drawing shows the end of gastrulation, from the dorsal side (profile at the level of the dorsal axial mesoderm). Hox expressing tissue (NOM (NO and I) and, late in gastrulation neurectoderm (N)) is represented by different colours, each of which represents a different hox code. Initially, the coloured bar represents the broken ring of NOM in the wall of the embryo. The later internal coloured blocks at the dorsal side of the embryo represent the involuted NOM mesoderm. The coloured blocks next to them in the wall of the embryo represent the overlying neurectoderm, which also comes to express hox genes. Hox expression codes are copied from the gastrula mesoderm to the neurectoderm. The SO is shown only in the last drawing, as the heavy median black line. By this stage, it has become the notochord and a head mesodermal portion. The first 5 drawings represent paraxial profiles, where the organiser is not available. The black dotted line in the last drawing depicts the sphere of influence of the SO. N: neurectoderm, NO: non-organiser mesoderm; S,: Spemann organiser; A: Anterior; P: Posterior; L: Left; R: Right. N nonorganiser; S Spemann organiser. The white arrows reflect directions of cell movement flow. To dorsal, anterior and internal(drawings 1 and 6). -There is a collinear time sequence of hox expression in non involuted non-organiser mesoderm (NOM) in the gastrula (depicted by the spectral sequence of colours). -During gastrulation involution movements continuously bring populations of cells from the NOM into the inside of the embryo, where their current Hox code is transiently stabilised. See stack of blocks of different colours, reflecting a history of the collinear hox mesodermal time sequence, in the internal involuted mesoderm. -Stable (ectodermal) Hox expression is induced by a combination of signals from the SO and the Hox expressing NOM. See corresponding blocks of sequential spectral colours in the gastrula's mesoderm and outer layer, reflecting a vertical transfer of the Hox codes from involuted mesoderm to overlying neurectoderm. A “Hox stripe” as part of the anterior–posterior Hox pattern is thus formed at the dorsal side [11, 14].

There is a temporally collinear sequence of Hox gene expression in the vertebrate gastrula’s non organiser mesoderm (NOM) and a spatially collinear pattern develops in the neurectoderm by the end of gastrulation [11, 13]. (Fig. **4A**) There is evidence that the NOM mesoderm’s Hox temporal collinearity is used to generate the primary Hox pattern of the embryo by time space translation. Each successive Hox combination in the temporally collinear mesodermal Hox expression sequence involutes into the gastrula at a specific time and appears to be copied to generate an identical combination at the correct place in the neurectoderm’s spatially collinear Hox pattern It has been shown that this signalling process is A to P time dependent. [11, 14].

## TIME SPACE TRANSLATION

Anterior–posterior positional information for the trunk in the vertebrate Xenopus is generated by sequential interactions between a timer in the early non-organiser mesoderm (NOM) and the Spemann organiser. The timer is characterised by temporally colinear activation of Hox genes in the early ventral and lateral mesoderm (i.e., the NOM) of the Xenopus gastrula. This early Hox gene expression is transient, unless it is stabilised by signals from the Spemann organiser [11]. The non-organiser mesoderm (NOM) and the Spemann organiser undergo timed interactions during gastrulation which lead to the formation of the anterior–posterior axis and stable Hox gene expression [11,14]. When separated from each other, neither non-organiser mesoderm nor the Spemann organiser is able to induce anterior–posterior pattern formation in the trunk. We deduced that convergence and extension movements during gastrulation continually bring more new cells from the NOM within the range of organiser signals that enable vertical signalling of the mesodermal Hox expression to a stable pattern in neurectoderm and thereby create patterned axial structures. In doing so, the age of the non-organiser mesoderm, but not the age of the organiser, defines positional values along the anterior–posterior axis [11]. The nature of the mechanism whereby organiser signals stabilise positional information is discussed below. We have evidence that the temporal patterning information from the non-organiser mesoderm is provided by the timed mesodermal Hox expression. The role of the organiser was investigated further and this turns out only to be the induction of neurectodermal tissue, which is a substrate for Hox expression, while non induced gastrula ectoderm is not [45]. Apparently, development of a stable axial hox pattern requires neurectodermal hox patterning. Fig. (**4B**) (14). The situation above is described for the amphibian Xenopus [11, 14], about which there is the most information, but as far as evidence is available, all vertebrates so far examined seem to have a similar situation, with Hox temporal collinearity during gastrulation. [8, 13, 15].

### The Basis of Hox Temporal Collinearity

**Fig. (5). Some facts and ideas about Hox colinearity.**
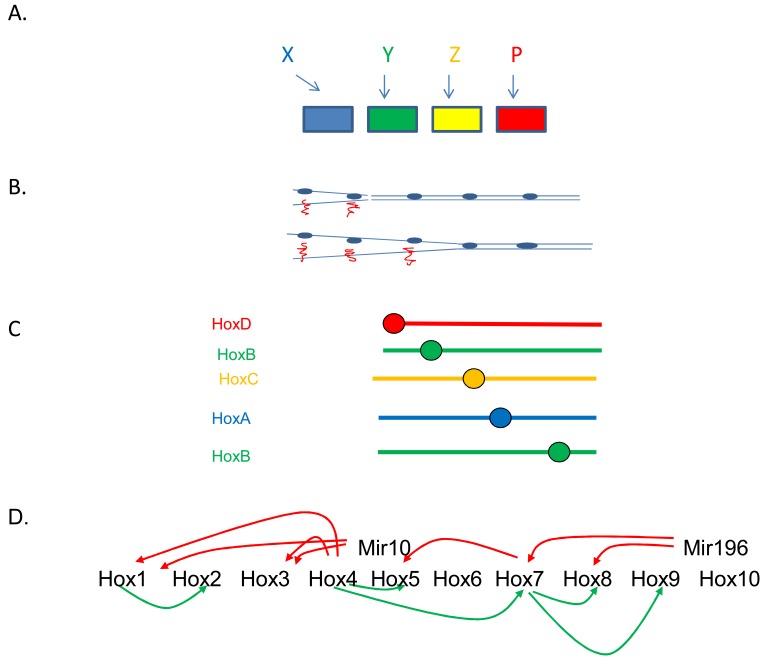
**A**. Upstream mechanism needed to generate spatial collinearity. In the case that there is no explicit colinearity mechanism, an individual input is needed to turn on each Hox gene to ensure it is expressed at exactly the right axial position. The inputs concerned are going to need an axial pattern themselves. This kind of mechanism is used in Drosophila, where the gap genes and segmentation genes provide the spatial inputs. Gap genes specify the primary axial positions where the Hox genes are expressed and segmentation genes, the Hox genes themselves, polycomb group genes and cofactors like teashirt refine this information, restricting Hox expression by specific segment boundaries. In this situation, the Hox genes thus do not provide the primary axial patterning information. They are secondary. It is likely that this kind of mechanism is general in invertebrates, which usually have no temporal colinearity or colinearity mechanism and have had to evolve an ad hoc mechanism to generate spatial collinearity. Something like this may also occur in the vertebrate hindbrain, where the gastrula’s colinearity mechanism is presumably the primary patterning mechanism and hindbrain genes confirm or alter the patterning information. **B**. Progressive chromatin opening: the basic idea. This is an idea proposed by Duboule and colleagues to account for vertebrate temporal collinearity. The Hox complex chromatin opens from 3’ to 5’. This opening progressively permits Hox gene transcription, from 3’ to 5’. **C**. Hox interactions.What regulates vertebrate temporal collinearity? Not just chromatin opening, as proposed by Duboule. The different vertebrate Hox clusters are expressed with synchronous temporal collinearity in the gastrula. The X axis shows time, increasing downwards. The Y axis shows 3’ to 5’ position in a Hox cluster. The figure shows that genes in different clusters are included in the same, temporally collinear sequence. **D**. What may be involved here are cross interactions between different Hox genes. The figure shows some of the interactions between Hox genes that occur in the vertebrate gastrula. **E**. A biological clock (the somitogenesis clock) may ensure the timing of Hox temporal collinearity [61]. Periodic pulses of X Delta2 may induce expression of particular Hox genes in NOM mesoderm during gastrulation and later.

The findings above emphasize the importance of Hox temporal collinearity for vertebrate development. They raise the question: what is the mechanism of vertebrate Hox temporal collinearity. This is unknown. It is widely believed that this is mediated by progressive opening of Hox complex chromatin, from 3’ to 5’ and/or by global enhancers that co-ordinately regulate the expression of the clustered Hox genes in a Hox complex. There is some evidence for this view [16, 17, 18, 47,48] (Fig. **5B**). However, the temporally collinear sequence of expression of the vertebrate Hox genes during gastrulation integrates members of the different vertebrate Hox clusters, which thus have synchronised temporal collinearity [11], see below and Figs. (**4** and **5C**). This means that chromatin opening is not an adequate explanation and that trans interactions (and since we are dealing with a multicellular mass), intercellular signals must be involved. It is not known what these trans acting factors and intercellular signals are. A possibility is that interactions among the Hox genes themselves are involved. There is a Hox collinearity property called posterior prevalence, whereby posterior Hox genes suppress function of and also actually repress transcription of and destabilise mRNA of more anterior Hox genes [1,19-26]. Posterior prevalence is important for vertebrate development [4, 25,27]. It occurs during vertebrate gastrulation [26,28,29,31] and can involve Hox associated microRNA’s (Heimberg and McGlinn, this volume [58]. It is accompanied by another collinear property, whereby anterior Hox genes stimulate expression of more posterior ones during gastrulation [3, 28, 30, 31], (Fig. **5D**). We think that these properties are a very plausible basis for a part or the whole of the mechanism of Hox temporal collinearity.

## WHAT IS THE NATURE OF THE TIMER?

Temporal collinearity needs to be precise, so that correctly timed Hox expression can translate to the correct spatial pattern in the embryo. This precise timing may be generated by the Hox collinearity mechanisms as above. However, it can not be ruled out that there is also an input from a biological clock (Fig. **5E**). There is a category of mechanisms based on oscillating patterns of gene expression or oscillating ion fluxes or metabolism that deliver precise timed information. The most famous examples are: circadian rhythms, the cell cycle, yeast glycollytic oscillations, the heart rhythm and neuronal oscillators. At least one mechanism of this type is active at the same time and in the same place as early Hox temporal collinearity. 

This mechanism is the’somitogenesis clock’. This is an oscillator that interacts with changing gradients of axial morphogens to generate the spatially periodic axial pattern of the vertebrate somites (mesodermal segments) [49]. It precisely controls the timing of segmentation and the spatial frequency and number of the somites. The somitogenesis clock is known to be associated with Hox patterning. The most striking findings are firstly that Hox anterior expression boundaries are somite (segment) boundaries. They change dynamically with somitogenesis and are even sometimes repeated periodically at several successive somites. Secondly that Hox expression is regulated by somitogenesis regulators; FGF, XDelta2, and RBPJk [50, 51, 52].

The somitogenesis clock begins operating during gastrulation, at the same time as Hox temporal collinearity. It also occurs in the same tissue (the NOM mesoderm, most of which later becomes presomitic and lateral plate mesoderm, the somitogenesis clock tissues) [50, 53]. Upregulating the oscillatory somitogenesis clock gene XDelta2 [54,55] during gastrulation prematurely upregulates at least 3 Hox genes: Hoxd1, Hoxb4, Hoxc6 [50]. Downregulating XDelta2 during gatrulation prevents expression of at least these same three Hox genes [50]. In addition, there is a positive feedback from Hox genes to somitogenesis. Downregulating the Hox1 paralogue group prevents somitogenesis and prevents expression of XDelta2 [56].

We conclude that the somitogenesis clock could plausibly be the timer for Hox temporal collinearity [61].

## TIME SPACE TRANSLATION IN EVOLUTION

Hox complexes are metagenes. A whole complex can function to pattern a body axis, via collinear properties. A single Hox gene can not [32, 33]. The paragraphs above have detailed how Hox temporal collinearity leads to axial patterning in the main A-P axis of the vertebrate Xenopus. This is an example of Hox metagene function. Do Hox genes pattern the body axis in this way in all metazoan animals? The answer is clearly: no. This vertebrate mechanism depends on Hox temporal collinearity, which is a rare property among metazoan Hox complexes. Invertebrate metazoans generally have incompletely functional Hox complexes The hox complex can be split and/or disorganised or the Hox genes can even be largely dispersed in the genome. Despite this, invertebrate Hox genes generally retain spatial collinearity (according to the Hox genes’ ancestral identities) [46], but do not often show temporal collinearity, which is rare. We’ll examine the case in Drosophila, where most is known. Drosophila, like all invertebrates, has a single Hox complex. Drosophila Hox collinearity is disorganised and disintegrating. The Hox complex is broken in two, to make the anterior Antennapedia and posterior Bithorax complexes [33]. The break occurs in different places in different Drosophila species. The Antennapedia and Bithorax genes and complexes are very large, which should hinder coordinated control of the Hox genes [33]. 

The Drosophila Hox genes are transcribed in different directions and the Antennapedia complex contains intercalated aberrant Hox genes, eg. Ftz and Zen that have non Hox functions in Drosophila [34, 35] but are normal functional Hox genes in some other metazoa. All of these observations indicate that the Drosophila Hox cluster is disintegrating and not fully functional and it comes as no surprise that the spatial collinearity manifested by this Hox cluster is primarily imposed by external upstream regulators: the spatially ordered gap genes and segmentation genes among others [36-43] (Fig. **5A**). Hox interactions also play a regulatory role here but they are not the primary spatial cues. We conclude that a Drosophila Hox cluster is not a fully functional metagene, it has no internal mechanism to fully mediate the spatial collinearity of the Hox genes and no temporal collinearity and no time space translation. Another interesting evolutionary question is how animals achieve unusual bodyplans using the same Hox genes. Woltering (Woltering, this volume [60]) addresses this question for the elongated bodies of snakes and Caecilians. He reaches the surprising conclusion that rather normal spatial collinearity is maintained but that particular Hox genes change their functions to encode different axial positions. 

## CONCLUSIONS

*Hox* genes are upstream regulators in the developmental hierarchy that are of great importance for generating the bodyplan. They specify and differentiate between different zones along the main body axis. These genes show collinearity- clustering associated with acquisition of ordered properties within a *Hox* gene cluster- a spectacular phenomenon that has attracted much interest. A* Hox *cluster is actually a metagene. It, but not an individual *Hox* gene, can fulfil a developmental function- patterning the body axis. In Drosophila, and probably in all other invertebrates- the full potential of the* Hox *genes is not realised. The expression of each individual *Hox *gene is regulated by other spatially regulated genes and so *Ho*x collinearity is not used to pattern the main body axis. In vertebrates, temporal collinearity has developed and this is used to pattern the main body axis and develop spatial collinearity, by time-space translation. It is presently generally assumed that the mechanism of temporal collinearity is progressive 3’ to 5’ opening for transcription of *Hox *complexes. This may be important. However, we suspect strongly that collinearity is partly mediated by *Hox* gene interactions. This idea was already indicated by early investigations of posterior prevalence. We review new evidence that trans-acting factors and intercellular signals mediate vertebrate *Hox *collinearity; that these include interactions among *Hox* genes, including posterior prevalence. We propose that these *Hox* interactions have a role in generating *Hox* temporal and spatial collinearity as well as functional collinearity. We note also that an evolutionary explanation for collinearity actually probably obviates any requirement for a dedicated collinearity mechanism. We also note that the precise timing of Hox temporal collinearity may be ensured by an input from a biological clock- the somitogenesis clock. Our conclusions open new perspectives for research into the mechanisms underlying collinearity. Testing them will require a much more extensive investigation and description of early vertebrate *Hox* temporal collinearity.

## Figures and Tables

**Fig. (1). Hox gene phenotypes.**
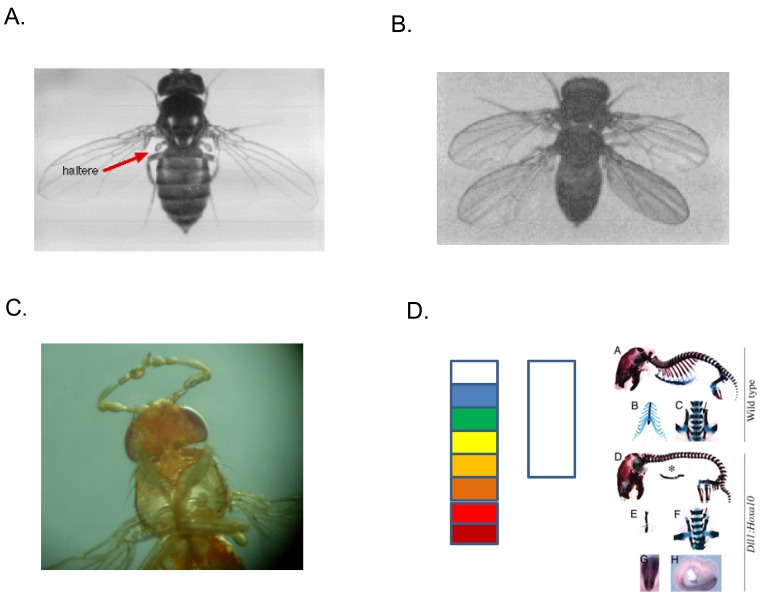
The function of Hox genes is defined by gain and loss of function phenotypes. The figure shows this in Drosophila and vertebrates. **A**. A wild type Drosophila fly This has two wings on the anterior thorax and two halteres (red arrow) on the posterior thorax. **B**. A four winged fly, caused by a loss of function mutation in ultrabithorax, a gene for posterior thorax [1]. The halteres are transformed to wings. **C**. Antennapedia mutation: mid thoracic legs replace antennae on the head, due to a misregulated gain of function mutation for the gene Antennapedia (a gene for mid thorax), leading to its expression in the head segments [2]. **D**. In vertebrates, mouse genetics has been bedevilled by the fact that there are 4 Hox clusters, with parallel functions. This once led to the erroneous idea that vertebrate Hox loss of function mutations have mild phenotypes. In fact, if you knock out all of the paralogues of a particular vertebrate Hox paralogue group (pg), or ectopically express a Hox gene this can give a dramatic phenotype. Left: diagram of wild type Xenopus hindbrain. This has 8 segments (rhombomeres) 2-8 each express a different combination of Hox genes and so have different identities, indicated by the different colours. 1 (white) expresses no Hox genes. Its identity is determined by the gene Gbx2. Middle: hindbrain in Xenopus where Hox pg1 has been knocked down using morpholinos. The hindbrain is drastically anteriorised to the identity of r1. It is also shorter (redrawn from [3]). Right: Skeletons of two mice. Above: wild type. Below, a mouse ectopically expressing HoxC10. The HoxC10 mouse is drastically different. For example, it lacks ribs [4]. The thoracic vertebrae are posteriorised to abdominal identity. This is because Hox pg10 controls the transition from thorax to abdomen, in the vertebral column.

**Fig. (2). Hox Spatial and Functional Collinearity.**
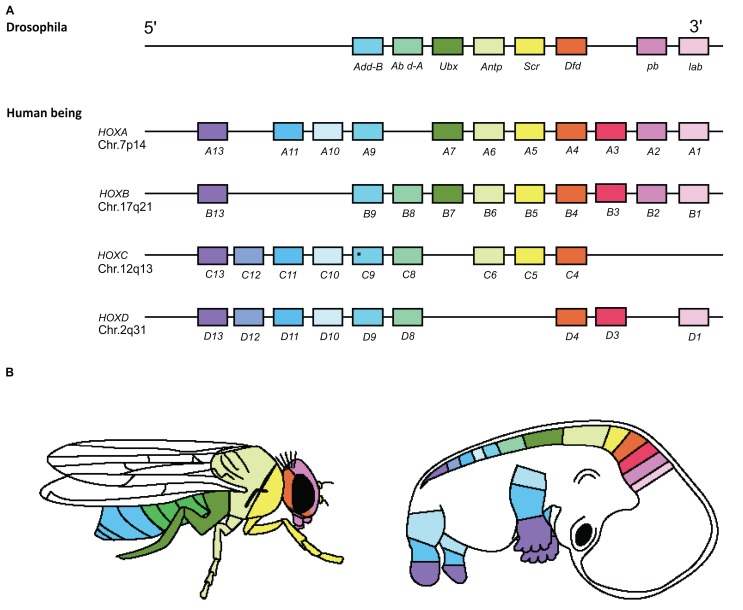
The four human and one Drosophila Hox complexes are homologues. The colour coding in Panels **A** and **B** shows the correspondence between the genomic order of Hox genes in the Hox complexes (**A**) and their spatial sequence of expression and action zones along the main body axis in Drosophila and human (**B**) [57].

**Fig. (3). The vertebrate A-P pattern is initiated during gastrulation.**
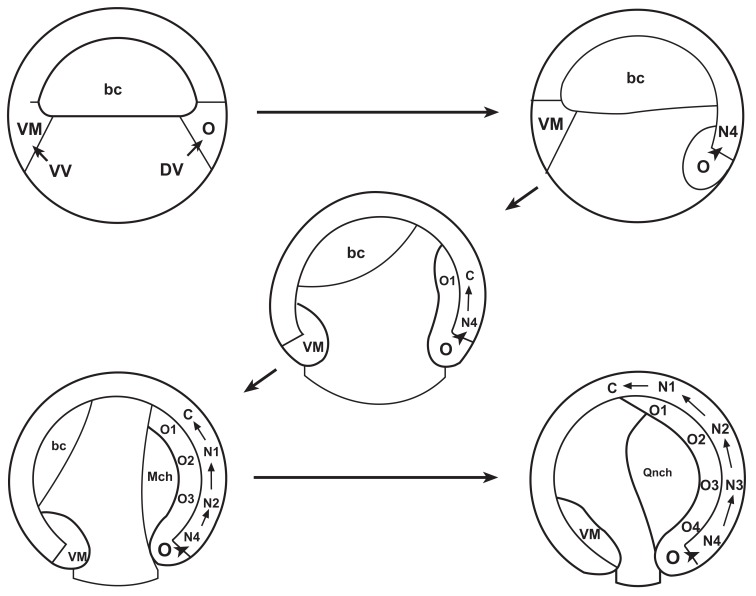
Drawings from an Amphibian embryo. From [9]. Top left, late blastula stage, just before gastrulation. Top right and the rest: successive stages through gastrulation, in the order indicated by the arrows. VM: ventral mesoderm, O: organiser mesoderm. VV: ventral endoderm DV: dorsal endoderm. Bc: blastocoels, arch: archenteron. O1, O2, O3, O4 successive A-P levels generated in the mesoderm during gastrulation, from anterior to posterior N1, N2, N3, N4: successive A-P levels in the developing central nervous system, generated in parallel with the mesodermal pattern by vertical signalling.
